# By the way, doctor: we've heard that sudden unexpected death in epilepsy is not a rare event. Do you know anything about this?

**DOI:** 10.1590/S1516-31802007000500013

**Published:** 2007-09-02

**Authors:** Fulvio Alexandre Scorza, Ricardo Mario Arida, Esper Abrão Cavalheiro

Epilepsy is the most common serious neurological condition. In developed countries, the incidence of epilepsy is around 50 per 100,000 people per year, and it is higher among infants and elderly people.^1,2^ The most commonly reported etiological factors are tumors, head injuries, stroke, genetic inheritance and infections of the central nervous system.^1,3^ In resource-poor countries, endemic infections such as neurocysticercosis and malaria seem to be major risk factors.^1^ Moreover, people with epilepsy are two to three times more likely to die prematurely than are those without epilepsy.^2^

The most common epilepsy-related category of death is sudden unexpected death in epilepsy (SUDEP). SUDEP is defined as sudden, unexpected death among epilepsy patients, with or without witnesses, not involving trauma or drowning, with or without evidence of a seizure and excluding documented *status epilepticus*, in which postmortem examination does not reveal any toxicological or anatomical cause of death.^4^ The information concerning the risk factors for SUDEP is conflicting, but the potential risk factors include: age, early onset of epilepsy, duration of epilepsy, uncontrolled seizures, seizure frequency, seizure type and number of antiepileptic drugs taken.^5^

The exact pathophysiological causes of SUDEP are unknown, but it is very likely that cardiac arrhythmia during and between seizures plays a potential role.^5^ Additionally, a recent study developed by our group evaluated the heart rate (*in vivo* and *in vitro*) and ventricular pressure *in vitro* of rats with epilepsy.^6^ Our results showed significant differences in the mean heart rate *in vivo* between the groups. In contrast, we did not find differences during *in vitro* experiments, thus suggesting the existence of central nervous system modulation of the heart, which could explain the sudden unexpected deaths among epilepsy patients.

Taken together, we believe that neurologists should be aware of SUDEP, and we recommend that epilepsy patients should undergo cardiological investigation. Strategies such as taking a detailed cardiovascular history to look for cardiovascular disease, symptoms and risk factors and examination of prior cardiac findings (electrocardiogram and echocardiogram) should be developed by cardiologists or general practitioners, in order to assess whether such strategies could prevent SUDEP, although this may require large population-based studies.
